# The Phenolic Compounds in the Young Shoots of Selected Willow Cultivars as a Determinant of the Plants’ Attractiveness to Cervids (Cervidae, Mammalia)

**DOI:** 10.3390/biology10070612

**Published:** 2021-07-02

**Authors:** Maciej Budny, Kazimierz Zalewski, Mariusz Jerzy Stolarski, Wiesław Wiczkowski, Adam Okorski, Robert Stryiński

**Affiliations:** 1 Polish Hunting Association, Research Station, Sokolnicza St. 12, 64-020 Czempiń, Poland; m.budny@pzlow.pl; 2 Department of Biochemistry, Faculty of Biology and Biotechnology, University of Warmia and Mazury in Olsztyn, Oczapowskiego St. 1a, 10-719 Olsztyn, Poland; robert.stryinski@uwm.edu.pl; 3 Department of Genetics, Plant Breeding and Bioresource Engineering, Faculty of Agriculture and Forestry, University of Warmia and Mazury in Olsztyn, Plac Łódzki 3, 10-719 Olsztyn, Poland; mariusz.stolarski@uwm.edu.pl; 4 Group of Chemistry and Biodynamics of Food, Institute of Animal Reproduction and Food Research, Polish Academy of Sciences, Tuwima 10, 10-718 Olsztyn, Poland; w.wiczkowski@pan.olsztyn.pl; 5 Department of Entomology, Phytopathology and Molecular Diagnostics, Faculty of Agriculture and Forestry, University of Warmia and Mazury in Olsztyn, Plac Łódzki 3, 10-719 Olsztyn, Poland; adam.okorski@uwm.edu.pl

**Keywords:** *Salix*, phenolic acids, flavonoids, salicylic compounds, wild animals, deer browsing

## Abstract

**Simple Summary:**

The presented study examined the phenolic acids, flavonoids and salicylates contents in young, 3-month-old shoots of different willow (*Salix* spp.) cultivars. The contents of individual compounds were not identical and depended on the cultivar from which they were isolated. Then, the severity of foraging damage by deer to the analyzed willow cultivars was evaluated and correlated with the content of specific compounds from each analyzed group. The quantitative relationships of helicine and salicin from the group of salicylic compounds, and between ferulic, trans-cinnamic and synapinic acid from the group of phenolic acids, can determine the foraging attractiveness of willow shoots to cervids.

**Abstract:**

This study examined the phenolic acids, flavonoids, and salicylates contents in young, 3-month-old shoots (including the leaves) of willow (*Salix* spp.). The cultivars were selected based on experiments carried out previously in Poland on fodder and energy willows. It was found, using the HPLC-MS/MS method, that the willow cultivars analyzed from three experimental plots, contained nine different phenolic acids, five salicylates and nine flavonoids, including four flavanols (quercetin, kaempferol, taxifolin and isorhamnetin), two flavanones (prunin, naringenin), two flavones (luteolin, apigenin) and one flavan-3-ol (catechin). The contents of individual compounds were not identical and depended on the cultivar from which they were isolated. The *S. laurina* 220/205 and *S. amygdalina* Krakowianka contained the greatest amounts of phenolic acids. The lowest quantities of these compounds were found in the *S. viminalis* Tur, *S. pantaderana* and *S. cordata* clone 1036. The highest concentration of flavonoids in young stems was found in *S. fragilis* clone 1043. The *S. purpurea* clone 1131 contained the highest amounts of salicylic compounds. Based on the results obtained from all experimental plots, it was shown that there is a negative correlation between the extent of browsing damage and the content of helicine and salicin from the group of salicylic compounds. A similar analysis between the phenolic acid concentration and the degree of willow browsing showed a positive correlation, especially between ferulic, trans-cinnamic, and synapinic acid. A negative correlation was found between the concentration of protocatechic acid content and browsing by cervids.

## 1. Introduction

The willow, *Salix* spp., includes approximately 430–440 species and an unspecified number of natural and artificial hybrids [1]. The spectrum of willow occurrence is very wide. The willow can be found in Northern Europe, Asia and North America, as well as in the mountainous regions of China. It is primarily found in the cold and subarctic zone of the northern hemisphere, throughout Europe, in Asia (including in mountainous parts of China), North America and Southern Africa. Few species are found in South America [2,3]. In Poland, 31 willow species grow in the wild: 25 native and six alien species. Numerous hybrids that are often difficult to identify also can be found (approximately 125) [4]. The natural habitats of willows in Poland include *inter alia* Żuławy Wiślane and the Biebrza, Bug, Narew or Warta River valleys. In principle, all willow species are pioneer plants characterised by high ecological plasticity. The large number of species and the morphological diversity of the *Salix* spp. plants offer many possibilities for their use. The willow (*Salix* spp.) includes very diverse, fast-growing species used for the production of biomass and generation of bioenergy [5,6,7,8,9,10,11,12,13,14], the production of second-generation biofuels [15,16], phytoremediation [17] and the control of erosion in the areas under risk [18]. This diversity is mainly due to the unique willow characteristics, including the high growth rate and extensive root system, as well as the ability to adapt to extreme environmental and soil conditions. The high ecological plasticity provides the possibility for the application of willows in making use of dry areas and reclaiming devastated areas. Willow plantings can form specific buffer zones around water bodies, landfills, sewage treatment plants and other devastated areas. It can also be used as raw material for the chemical industry (cellulose). Due to the high salicylate and tannin content, the willow bark is a potential raw material for the pharmaceutical and chemical industries. What remains after the extraction of valuable bioactive compounds can be used for energy-generating purposes [14,19]. The main objective is to maximise the use of willow biomass as higher value-added bioproducts to obtain medicines, cellulose, hemicellulose and lignin and as a fodder form. Moreover, shrubby forms of the willow have been successfully used for centuries in water engineering (fascine). On the other hand, the wattle forms of the willow are the raw material to produce woven furniture and basketry products.

It should be stressed, however, that other aspects of willow cultivation are also important, which should be considered for the cultivation of forageable willows. Early flowering willow plants are an important source of food for bees, bumblebees and other insects. Willow plantations provide a good habitat for wild animals and birds. Studies on bird populations have concluded that more than 30 bird species could be found in energy willow shrubbery. In Denmark, small plantations of fast-growing shrubs have been established with no commercial purpose, only for the breeding of gamebirds. Therefore, a new trend in willow use may be the application of its plantations as a foraging source for cervids. Research in this field has been carried out since as early as 1962. In Poland, such experiments were also conducted [20,21,22,23,24]. In the last three decades, experiments with foraging willow have been managed by Drogoszewski [25,26,27]. As a result of the research conducted, the team recommended several willow varieties and cultivars for cultivation, which have been largely implemented. However, observations from other plantations (energy willow), in other regions of the country indicated that, depending on the abundance of the stem-based food and the number of cultivars available to the deer, other attractive forms of willow can be selected for animals to reduce game damage in forest crops and post-agricultural plantings. Of the several dozen cultivars in Poland, and of the several dozen wild forms, deer intensely forage only on certain forms, which leads to the conclusion that this is determined by the chemical composition of the bark and phloem, as well as by the composition of age, from 3 to 5-month-old stems foraged most willingly in the spring and summer.

So far, many studies have been published on the chemical composition of willow shoots of various species and cultivars. A significant part of these works concerns energy crops, while the remaining works concern the therapeutic use of compounds contained in willow sprouts (bark). There are no studies on the relationship between shoots biting by game and their chemical composition, except for one that we published earlier [28]. Due to that, the primary aim of this study was the analysis of phenolic compounds content in selected willow cultivars.

## 2. Materials and Methods

### 2.1. The Plant Material

The nursery plot at the Research Station of the Polish Hunting Association in Czempiń (Wielkopolskie Voivodeship, Poland) was established using cuttings obtained from the Resko Forest Inspectorate (*S. fragilis* Kamon, *S. amygdalina* Dunajec, *S. amygdalina* Krakowianka), as well as cuttings from experiments carried out at the experimental stations of University of Warmia and Mazury in Olsztyn, located in Bałdy, Poland (*S. fragilis* clone 1043) and in Obory, Poland (the other species used for the analyses).

The individual cultivars were numbered, which is presented in Table 1. For the 3 cultivars, markings X_1_ to X_3_ were introduced, since there are no results for them regarding the degree of foraging by deer, and that they are not considered on figures concerning foraging in connection with the content of compounds under analysis.

At the end of July 2017, ten 3-month-old shoots were obtained from each willow cultivar (3 replicates) in the nursery plot in Czempiń, Poland, and analysed for the phenolic acid, flavonoid and salicylate contents.

### 2.2. The Samples Preparation for HPLC-MS/MS Analysis

For the high-performance liquid chromatography and tandem mass spectrometry (HPLC-MS/MS), the upper parts (10 cm tips) of the approximately 3-month-old shoots were collected (3 replicates from each willow cultivar).

MS grade reagents, including acetonitrile, methanol, water and formic acid were purchased from Sigma Chemical Co. (St. Louis, MO, USA). Diethyl ether (Et_2_O), hydrochloric acid (HCl) and sodium hydroxide (NaOH) were obtained from POCH S.A. (Gliwice, Poland). Standard compounds (phenolic acid, flavonoids, salicinoids) were from Sigma Chemical Co. (St. Louis, MO, USA).

The obtained willow material was dried for 96 h at 45 °C and ground in an impact mill. The samples were then extracted with petroleum ether in a Soxhlet extractor for 4 h. Ether extracts were discarded. After evaporating ether residues in the same apparatus, the samples were extracted for 6 h with methanol, which was evaporated in a vacuum evaporator (temperature of 45 °C), and the residue was stored in a freezer in liquid nitrogen.

Compounds (free and those released from soluble esters and soluble glycosides), were isolated from the extracts according to the followed method. The residue obtained was dissolved in methanol and centrifuged (13,200× *g* at 4 °C, 20 min). Next, after evaporation of the methanolic extract (0.1 mL) to dryness under nitrogen at 35 °C, the residue was dissolved in water (0.4 mL, adjusted to the pH of 2 with 6 M HCl), free compounds were extracted 3 times by 1 mL of Et_2_O using vortexing (30 s) and sonication (30 s). After centrifugation for 5 min (5000× *g* at 4 °C), the ether extract was collected and evaporated to dryness under nitrogen at 35 °C. In the second step, 1 mL of 4 M NaOH was added to the remaining extract and the mixture was placed in a nitrogen atmosphere and hydrolysed for 4 h at room temperature. After acidification to a pH of 2 using 6 M HCl, compounds liberated from soluble esters were extracted 3 times by 1 mL of Et2O using vortexing (30 s) and sonication (30 s). After centrifugation for 5 min (5000× *g* at 4 °C), the ether extract was collected and evaporated to dryness under nitrogen at 35 °C. In the third step, 0.2 mL of 6 M HCl was added to the remaining extract and the mixture was hydrolysed for 1 h at 100 °C. After adjusting to the pH of 2 using 8 M NaOH, compounds released from soluble glycosides were extracted three times by 1 mL of Et_2_O using vortexing (30 s) and sonication (30 s). After centrifugation for 5 min (5000× *g* at 4 °C), the ether extract was collected and evaporated to dryness under nitrogen at 35 °C. All samples with dry residue obtained after ether evaporation were dissolved in 0.1 mL of 80% methanol, centrifuged (13,200× *g* at 4 °C, 20 min) and injected to an HPLC-MS/MS system.

### 2.3. HPLC-MS/MS Analysis

Aliquots (2 µL) of extracts were injected into an HPLC system (LC-200, Eksigent, Dublin, CA, USA) equipped with a dual-channel pump, column oven, autosampler (set at 4 °C) and system controller link to an Analyst 1.5.1 system. Chromatographic separation was conducted with a HALO C18 column (2.7 µm particles, 0.5 × 50 mm, Eksigent, USA) at 45 °C at a flow rate of 15 µL/min. The elution solvents were A (water/formic acid; 99.05/0.95; *v*/*v*) and B (acetonitrile/formic acid, 99.05/0.95, *v*/*v*). The gradient was used as follows: 5% B for 0.1 min, 5–90% B in 1.9 min, 90% B for 0.5 min, 90–5% B in 0.2 min and 5% B for 0.3 min. For HPLC-MS/MS analysis, a QTRAP 5500 ion trap mass spectrometer (AB SCIEX, USA) was connected to the Eksigent LC200 via an ESI interface. Optimal ESI-MS/MS conditions and data for the calibration curve for phenol separation by HPLC are presented in the Supplementary Materials Tables S1 and S2.

### 2.4. Foraging Plots

**Figure 1:**
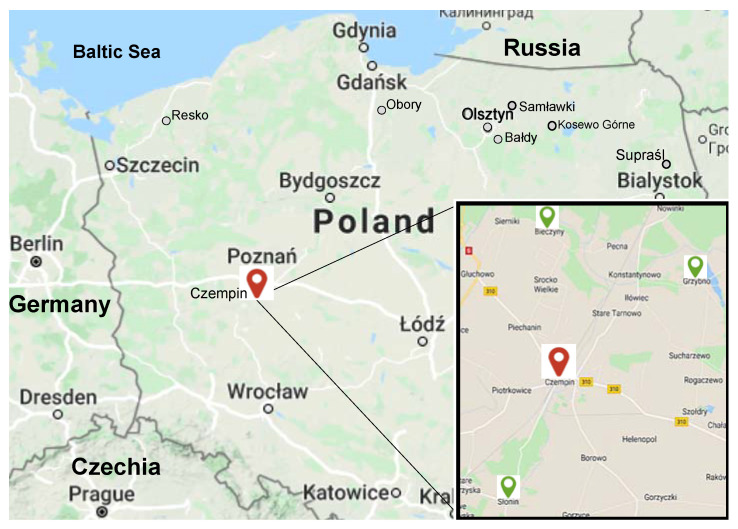
The location of foraging plots. The exact geographic coordinates are given in the text.

The willow nursery plot in Czempiń, Poland was cut in the spring of 2016 and in early March 2017, and one-year-old shoots were used to establish three experimental foraging plots near Grzybno, Poland (X: 482950.30; Y: 351700.42), Bieczyny, Poland (X: 484298.21; Y: 345303.04), and Słonin, Poland (X: 475485.18; Y: 344974.35) (Figure 1).

The characteristics of soil on a nursery plot and foraging plots was previously described by Budny et al. [28]. The willows on foraging plots were planted as described before [28].

### 2.5. Foraging Damage Assessment

In the fall of 2017 and 2019, the damage caused by wild animals in foraging plots (after fencing had been removed) was assessed on a 5-point scale proposed by Bukiewicz [20], with some modifications described by Budny et al. [28].

When assessing the foraging damage, 5 repetitions were made, each with 20 consecutive seedlings in a row. For cultivars with less than 200 plants per plot (cultivars No. 2, 3, 5, and 9), all seedlings were assessed. The bite size was assessed as a percentage, e.g., 10% bite-size means that 10% of the plants within the cultivar was eaten by deer species.

### 2.6. Statistical Analysis

The results were processed statistically in the Statistica program (v. 13.1, Dell Inc., Tulsa, OK, USA). The severity of foraging damage to the analyzed willow cultivars was evaluated by analysis of variance and Tukey’s test. The Spearman’s rank was used to calculate the correlation coefficients between of the extent of foraging and specific compounds in the evaluated willow cultivars.

## 3. Results

### 3.1. The Phenolic Acids Content

**Figure 2:**
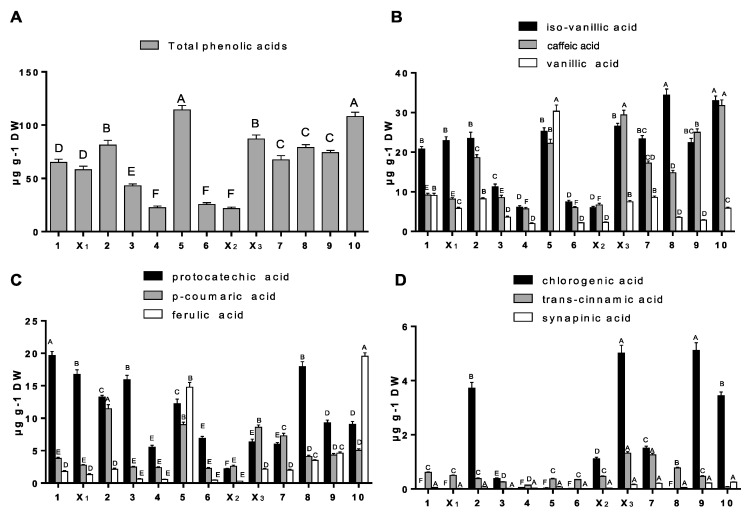
The concentration of total phenolic acids (**A**), including iso-vanillic, caffeic and vanillic acid (**B**), protocatechuic, p-coumaric and ferulic acid (**C**), chlorogenic, trans-cinnamic and sinapinic acids (**D**). The values are means (*n* = 3) ± SD. Symbols: 1–10–numbers of cultivars as in Table 1.

The data presented in Figure 2, concerning the phenolic acid content in the analysed material, indicate that all willow samples contained nine different phenolic acids. The total content of these acids and the contents of individual acids in analysed young shoots varied greatly (Figure 2).

In total, the highest phenolic acid content was found in *S. laurina* 220/205 and *S. amygdalina* Krakowianka, and a slightly lower content was noted in cultivars no. 2, and X_3_ (Figure 2A). The Tur cultivar (cultivar X_2_) and *S. pentederana* (cultivar 4) contained the least acids. The same figure shows three groups of phenolic acids grouped according to the number found in the analysed cultivars. The first group (Figure 2B) comprises acids found in the largest quantities (*iso-*vanillic acid, caffeic acid and protocatechic acids). Large amounts of these acids were in *S. laurina* 220/205 and *S. amygdalina* Krakowianka (77.75 and 70.60 µg/g of dry matter: DM, respectively), and the smallest in the *S.* *viminalis* Tur cultivar (14.98 µg/g DM, Figure 2B).

The contents of the acids found in amounts ranging from 0.56 to more than 30 µg/g DM are presented in Figure 2C. The largest amount of these three acids was observed in *S. laurina* 220/205 (cultivar 5), with a particularly high ferulic acid content. High values were also observed in the stems of *S. amygdalina* Krakowianka (cultivar 10). The *S. pantaderana* and the *S.* *viminalis* Tur cultivars (no. 4 and X_2_) were characterised by the smallest quantity of the analysed acids in young stems (Figure 2C). As shown in Figure 2D, the majority of analysed willow cultivars contained low concentrations of chlorogenic acid, but its quantities in individual cultivars varied greatly. In the last group of acids, chlorogenic acid was found in largest quantities in cultivars of *S. cordata* Ekotur (no. X_3_) and *S. amygdalina* Dunajec (no. 9). The quantities of sinapinic acid in all samples were very small, occasionally at the detection limit, and did not exceed the value of 0.25 µg/g DM (Figure 2D).

### 3.2. The Flavonoids Content

Nine different flavonoids, including four flavanols (quercetin, kaempferol, taxifolin and isorhamnetin), two flavanones (prunin, naringenin), two flavones (luteolin, apigenin) and one catechin (flavan-3-ols) were found in all the stems of the analyzed willow cultivars. The flavonoids concentrations are presented in Table 2.

The flavonoids amount in individual samples was very diversified and ranged from trace amounts (naringenin, taxifolin) to considerably high concentrations (catechin). From among the 13 analyzed willow cultivars, the cultivar *S. fragilis* 1043 (no. 2) stood out in respect of the high concentration of total flavonoids, mainly due to the high concentration of catechins in its stems. Significantly lower flavonoids amounts, but still relatively high compared to other willows, were reported in the *S. cordata* Ekotur (cultivar X_3_) stems. The lowest concentrations of the described compounds (the total concentration of all the analyzed flavonoids) were found for the cultivars of *S. amygdalina* 1102 (no. 8) and *S. cordata* 1036 (no. 6). When analyzing the content of the individual flavonoids in the willow samples, it was demonstrated that naringenin and taxifolin occurred in the lowest amounts, below 0.20 µg/g DM (at the detection threshold), and in some samples the concentration of pruning was also at a low level. The amount of catechin in young shoots was the most diversified and ranged from 3.04 in *S. amygdalina* 1102 (no. 8) to 572.73 µg/g DM in *S. fragilis* 1043 (no. 3). The content of the other flavonoids in the analyzed samples was also very diversified and depended on the cultivar (Table 2).

### 3.3. The Salicynoids Content

Of the five salicylate compounds detected and determined quantitatively in the stems of the analysed willow cultivars, the amounts of helicine and picein exceeded the value of 5% in only two varieties. The *S. purpurea* clone 1131 (cultivar X_1_) and *S. amygdalina* 1102 (cultivar 8) contained the highest glycoside content. This was because, as compared to other willow, the cultivars in question contained large amounts of salicin and saligenin. It should be noted that the analyzed glycoside content in individual willow cultivars varied greatly and that these differences in certain cases were even more than 82-fold (salicin) and 60-fold (saligenin) (Table 3).

The total contents of phenolic acids, flavonoids and salicylate compounds in young stems of selected willow cultivars in the spring of 2017 was also calculated (Table 4).

### 3.4. Foraging Experiment

In the spring of 2017 and 2019 in the districts, where the foraging plots were established, the inventory was performed. The number of individuals for each deer species is given in the Table 5. The data shows that the degree of damage to willows in the plot in Słonin was mainly influenced by roe deer, which were often observed during foraging on this plot. The plot in Bieczyny was visited by deer and roe deer. On a plot in Grzybno, mainly roe deer and, occasionally, deer foraged.

**Figure 3:**
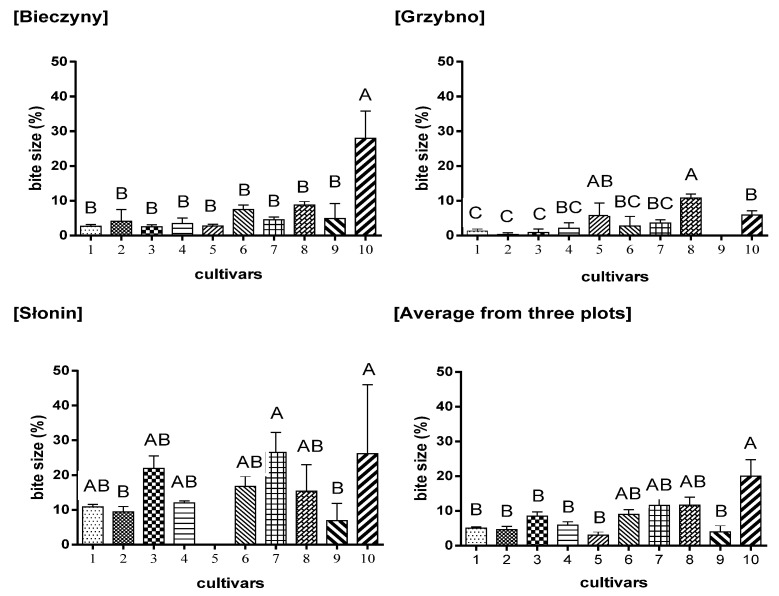
The severity of foraging damage to the analyzed willow cultivars. Bars with the same letters did not differ significantly (*p* ≤ 0.01). Symbols: 1–10–numbers of cultivars as in Table 1.

As shown in Figure 3, the *S. amygdalina* Krakowianka cultivar (no 10) was the most attractive for animals. From three plots, almost 30% of this willow cultivar was eaten by different deer species. The other willows were damaged to an extent determined by the species of animals living in the vicinity and by the surrounding habitat (around 10% of plants were damaged).

The analysis of the damage of the cultivars on individual plots (Figure 3), together with the number of animals (Table 5) leads to the conclusion that the number of animals defined as deer, influences the degree of damage in individual plots but does not affect the foraging of individual cultivars. The greatest damage was observed in Słonin and Bieczyny (Figure 3), where the greatest number of deer was also noted (Table 5).

Based on the results obtained from all test plots, it was shown that there is a negative relationship between the content of helicine and salicin vs. the extent of browsing damage in the evaluated willow cultivars. The following correlation coefficients have been calculated as R = −0.21 and R = −0.22, respectively (Table 6).

Correlation analysis towards the concentration of phenolic acids and the extent of browsing damage revealed a positive correlation between willow browsing and acid content, i.e.,: ferulic (R = 0.39), trans-cinnamic (R = 0.35), synapinic (R = 0.38) and total phenolic acids content (R = 0.35) in the experimental plot in Grzybno. A negative correlation was found between the concentration of protocatechic acid content (R = −0.37) and browsing by cervids on the plot in Słonin (Table 7).

The statistical analysis covering all three localities simultaneously showed a weak positive correlation between the content of ferulic acid (R = 0.28) and synapinic acid (R = 0.28) and the level of browsing damage on willow shoots.

The analysis of Spearman’s rank correlation on the amount of flavonoids in willow shoots with deer browsing showed that all results with catechin in all plots were at the level of 0.0x, i.e., no relationship with browsing and the catechin content was found. The analysis of the remaining flavonoids also showed no statistically significant differences.

This could be transferred into the conclusion that the degree of browsing on individual cultivars depended on the chemical composition of their shoots, mostly on the content of phenolic acids and salicylic compounds.

## 4. Discussion

The damage caused to forest crops and older tree stands in Poland and Central Europe is well-documented and has serious economic significance. To date, no research has been undertaken into the issue of the size of the damage and the degree of foraging on young stems of individual species in forests vs. the chemical composition of this food foraged by cerevids. It needs to be considered that almost all tree and shrub species found in Europe are foraged by cerevids, but not only by them. There are known cases noted in the Wigierski National Park (North Poland), where European beavers, during a short period of time, felled 1.5 ha of the old-growth pine forest for reasons which are still unknown. The nutritional requirements of these two species are completely different, but such extreme cases of behavior indicate that no animal behavior is uniform in various habitats. Currently in Poland (in complete contrast to the actions undertaken by foresters in Austria), the most radical method for protecting forest crops against the damage caused by forest animals has been applied, i.e., fencing off [29]. It appears that the method has more opponents than supporters, and the different views of forest owners and the game shooting district tenants are a reason for multiannual disputes and conflicts [30,31]. The attempts undertaken to solve this problem have yielded no expected results [32]. Nowadays, it is not very clear as to what should be done about this problem. It is just not possible to fence off an entire forest [33]. The greatest damage in nurseries and tree stands is caused in the spring and early summer and not in the winter. Therefore, this study analyses young, non-woody 2.5 to 3-month-old stems since the demand for this type of food is the greatest in the spring period, when female cervids are in late pregnancy, and the males’ antlers are formed. Minerals, vitamins, carbohydrates, pro-health compounds and others are required in food.

The results obtained by the authors from chemical analyses of the willows selected for the study clearly indicate that individual varieties and cultivars in young, non-woody stems are characterized by varied contents of compounds, such as phenolic acids, flavanols and phenolic glycosides. Previously, we showed an identical relationship regarding the content of soluble carbohydrates in willow shoots [28]. To date, it has not been clearly demonstrated which of these compounds has a positive effect and which has a negative effect on the forage attractiveness for cervids. The analysis of the damage by cultivars on individual plots, together with the number of animals in hunting districts, leads to the conclusion that the number of animals defined as deer, influences the degree of damage in individual plots but does not affect the browsing of individual cultivars. The degree of nibbling of individual cultivars depended on the chemical composition of their shoots, which was proven using statistical analyses (Table 6 and Table 7).

The results concerning the content of phenolic acids in relation to the degree of browsing of individual cultivars obtained in our experiments do not give an unambiguous answer to the question: do the health-promoting effects of phenolic acids attract the game more than the taste of these phenols? It is known that phenolic acids give plant tissues a bitter and sour taste [34,35]. However, the statistical analyses presented in the “Results” section showed a correlation between the content of several phenolic acids in the willow tissues and the degree of browsing damage, at least of ferulic and sinapinic acid. This means that deer are not always guided by taste when choosing food. On the other hand, when giving opinions on the results, the condition of the environment (e.g., droughts) and the availability of food for deer in the vicinity of the established experimental plots should be considered. These conditions in the vicinity of the three analysed experimental plots were not very good.

Numerous data provided in publications indicate that for humans (no cerevids were indicated), salicin and salicortin are the bitterest, while other glycosides are mentioned in the group of moderately bitter compounds (saligenin, populin, grandidentatin, salireposide and isosalipurposide). Do the taste buds of cervids transmit the taste stimuli to the nervous system similarly as in humans? There is no answer to this question thus far. A statistical analysis of the current study shows that salicin and helicine have a negative effect on the forage attractiveness of the stems of analysed willow cultivars for cervids. This statement is consistent with the data published by Julkunen-Tiitto and Gebhard [36], who claim that these salicylates (+tremulacin) not only have a bitter taste but also irritate the mucous membranes. This discourages potential consumers from eating this type of food. The obtained results, however, need to be looked at. For example, catechin, whose large amounts were found in young stems of *S. fragilis* clone 1043, is a flavonoid commonly found in plants (green tea, fruits, cocoa beans) and a natural antioxidant. It has antibacterial, antiviral, antifungal, anti-inflammatory, anticoagulant and antioxidant effects and lowers blood pressure. Catechin prevents cancers, and is used in the treatment of neoplasms, diabetes mellitus, cataracts, obesity etc [37,38,39]. In January 2020, in a stockyard in Czempiń and in a breeding stockyard of the Polish Academy of Sciences in Kosewo Górne, Poland, an initial nutritional experiment involving fallow deer and deer was carried out. Its results showed that catechin is a substance that attracts cervids and increases the intake of stem-based food (unpublished data). Moreover, based on multiannual observations carried out on a willow plantation, it was found that two willow forms of *S. purpurea* were not foraged by wild animals. The most attractive food for cervids (roe deer, fallow deer) in this study was *S. amygdalina* clone 1102, and *S. amygdalina* Krakowianka, while in other experiments carried out on plantations in Zachodniopomorskie Voivodeship, it was *S. cordata* “Nicholsoni” Purpurescens and *S. pantaderana* (unpublished data).

The introduction of forage willows to the forage resources of cervids is only a partial method for reducing losses in young forest crops. The method has a health-related aspect as well. These plants contain phenolic compounds in their tissues, including salicin and other phenolic glycosides, which have anti-inflammatory, antipyretic, antifungal and analgesic effects. Flavonoids and tannins also exhibit health-enhancing effects [40]. These compounds improve the health status and condition of animals, even though these claims have not been widely supported by scientific research [41].

The considerations about what deer and roe deer prefer in natural food, and why certain tree species are more attractive for them than others, should be considered in two basic aspects. The first aspect is the availability of food in the environment where the animals live. A mixed coniferous forest offers everything that the deer require throughout the year (various tree species, including oak, undergrowth with berries, mosses, etc.). The opposite in the forage resources of cervids is a dry coniferous forest and fresh forest, which offer no undergrowth. Therefore, the greatest density of cervids in Europe is found in mixed coniferous forests.

Due to this, the greatest browsing damage caused by cervids occurs in plantings and young tree stands in mixed coniferous forests. To reduce the volume of losses and to solve this problem, numerous actions have been undertaken. One of them was to strive to enrich the forage resources of cervids by planting foraging trees (willow and poplar). At this point, it is worth mentioning a study by Moore et al. [42] on experiments conducted in New Zealand (Wairarapa district), which is very much distant from Poland. The experiment involved a partial supplementation of the meat cattle diet during a drought with nothing else but foraging willow crops. The experiments demonstrated a positive effect of the diet supplementation in the form of willow biomass on the condition and meat weight gains of cattle in the stressful period of drought. Therefore, a question arises as to whether the stressful period of winter and early spring in Poland and the neighboring countries for cervids can be compared with the drought in New Zealand and the meat cattle grazing in that period? It appears that it is partially comparable because both groups of animals are ruminants, which eat young willow stems or parts (bark + phloem, the so-called “stripping”). If meat cattle, which are not accustomed to such a diet, are able to effectively use willow as food, then such food can, and should be, a component of a diet for cervids living in the wild.

Another method for reducing damage has been the use of chemical preparations (the application of repellents onto the apical shoot), wrapping sheep’s wool or hemp oakum around the apical bud (oakum wrapping), the protection of larch trees against the deer by driving three stakes into the ground, the use of plastic (tekpol) shields and fencing off for a period of a minimum of 10 years.

To date, it has not been determined what guides cervids under the conditions of the possibility for choosing food. It is known that they have a much better sense of smell and recognize many more flavors in a considerably lower concentration than do humans [43,44,45]. Is the foraging attractiveness determined by the amounts of phenols, tannins, soluble carbohydrates and waxes (by a single factor), or by a combination of components? To date, it has not been understood. It is sufficient to mention that other attractively tasting compounds are found in the world of plants, e.g., very sweet proteins, such as thaumatin, miraculin, brazzein, pentadin and others [46]. To our knowledge, there are no scientific reports indicating that such peptides and proteins are found in willow.

The results obtained indicate a correlation between the degree of foraging on young shoots of willows and the specific phenolic acids, and salicylates contained in them in the spring and summer. In the winter, even when one-year-old stems are already woody, the bark contains more biologically active substances than the leaves and young stems in the spring and summer, which indicates the need for fencing off the foraging plots at the appropriate time, and their proper management [47].

## 5. Conclusions

The presented study examined the phenolic acids, flavonoids and salicylates contents in young, 3-month-old shoots (including the leaves) of different willow (*Salix* spp.) cultivars. The contents of individual compounds were not identical and depended on the cultivar from which they were isolated. The results obtained indicate a correlation between the degree of foraging on young shoots of willows and the specific phenolic acids, and salicylates contained in them. The quantitative relationships of helicine and salicin from the group of salicylic compounds, and between ferulic, trans-cinnamic, and synapinic acid from the group of phenolic acids, can determine the foraging attractiveness of willow shoots. Moreover, based on the conducted research, it can be concluded that the cultivation of *S. amygdalina* clone 1102, and *S. amygdalina* Krakowianka, most often eaten by cervids, as a barrier, may contribute to the reduction of losses caused by these animals in areas where forests are weaned.

## Figures and Tables

**Table 1:** The cultivar numbers used later in the work in the tables and figures.

The Willow Species	Cultivar No.
*S. purpurea* 1126	1
*S. purpurea* 1131	X_1_
*S. fragilis* clone 1043	2
*S. fragilis* Kamon	3
*S. pantaderana*	4
*S. laurina* 220/205	5
*S. cordata* 1036	6
*S.viminalis* Tur	X_2_
*S. cordata* Ekotur	X_3_
*S. amygdalina* Triandra 1045	7
*S. amygdalina* 1102	8
*S. amygdalina* Dunajec	9
*S. amygdalina* Krakowianka	10

**Table 2:** The flavonoid content in young stems of selected willow varieties and cultivars from the Czempiń plantation in the third 10-day period of May 2017 (µg/g DM). The mean values from three replicates were supplemented with a standard deviation. The numbers of cultivars as in Table 1.

Flavonoids										
Cultivars No.	Taksifolin	Quercetin	Isorhamnetin	Apigenin	Kaempferol	Luteolin	Catechin	Naringenin	Prunin	Total Flavonoids Content
1	0.09 ^F^ ± 0.00	4.40 ^GH^ ± 0.13	2.78 ^F^ ± 0.12	4.37 ^F^ ± 0.16	0.85 ^C^ ± 0.03	50.86 ^C^ ± 1.49	5.08 ^G^ ± 0.16	0.11 ^B^ ± 0.01	1.11 ^E^ ± 0.05	69.65 ^E^ ± 4.51
X_1_	0.24 ^B^ ± 0.01	6.20 ^FG^± 0.24	0.59 ^F^ ± 0.03	3.46 ^FG^ ± 0.13	0.77 ^CD^ ± 0.02	42.62 ^D^ ± 1.32	7.92 ^G^ ± 0.28	0.08 ^C^ ± 0.01	4.27 ^A^ ± 0.22	65.25 ^EF^ ± 2.47
2	0.19 ^C^ ± 0.00	12.81 ^E^ ± 0.26	22.97 ^D^ ± 0.58	26.94 ^B^ ± 1.02	1.17 ^B^ ± 0.03	62.29 ^B^ ± 2.40	572.73 ^A^ ± 17.70	0.03 ^DE^ ± 0.01	1.53 ^D^ ± 0.05	700.66 ^A^ ± 31.34
4	0.08 ^G^ ± 0.00	0.94 ^IJ^ ± 0.03	1.00 ^F^ ± 0.03	2.44 ^G^ ± 0.08	0.23 ^HI^ ± 0.01	14.33 ^GH^ ± 0.72	119.03 ^E^ ± 3.07	0.04 ^DE^ ± 0.01	0.68 ^F^ ± 0.03	138.77 ^D^ ± 3.88
4	0.10 ^F^ ± 0.00	0.57 ^I^ ± 0.02	0.04 ^F^ ± 0.00	4.04 ^EF^ ± 0.13	0.65 ^EF^ ± 0.02	35.86 ^E^ ± 1.14	46.98 ^F^ ± 2.11	0.19 ^A^ ± 0.01	2.28 ^C^ ± 0.16	90.71 ^E^ ± 4.26
5	0.10 ^F^ ± 0.00	0.85 ^IJ^ ± 0.03	1.90 ^F^ ± 0.05	7.31 ^E^ ± 0.19	0.51 ^G^ ± 0.02	26.69 ^F^ ± 0.85	30.87 ^F^ ± 1.06	0.01 ^E^ ± 0.00	0.15 ^G^ ± 0.01	68.39 ^EF^ ± 3.43
6	0.17 ^D^ ± 0.01	2.97 ^HI^ ± 0.08	1.51 ^F^ ± 0.07	2.35 ^G^ ± 0.07	0.33 ^H^ ± 0.01	2.31 ^I^ ± 0.11	8.09 ^G^ ± 0.23	0.05 ^D^ ± 0.00	0.80 ^F^ ± 0.02	18.58 ^G^ ± 0.72
X_2_	0.03 ^I^ ± 0.00	8.07 ^F^ ± 0.23	16.10 ^E^ ± 0.36	10.24 ^D^ ± 0.58	0.12 ^I^ ± 0.01	1.77 ^I^ ± 0.07	32.57 ^F^ ±0.92	0.01 ^E^ ± 0.00	0.05 ^G^ ± 0.00	68.96 ^EF^ ± 2.83
X_3_	0.33 ^A^ ± 0.01	59.80 ^A^ ± 2.37	77.18 ^A^ ± 2.46	36.10 ^A^ ± 1.32	2.47 ^A^ ± 0.09	11.11 ^H^ ± 0.19	214.06 ^BC^ ± 7.15	0.02 ^DE^ ± 0.00	0.19 ^G^ ± 0.02	401.26 ^B^ ± 15.23
7	0.05 ^H^ ± 0.00	21.67 ^C^ ± 0.97	37.09 ^B^ ± 1.23	19.76 ^C^ ± 1.11	1.28 ^B^ ± 0.05	0.65 ^I^ ± 0.02	192.86 ^CD^ ± 6.79	0.01 ^E^ ± 0.00	0.06 ^G^ ± 0.00	273.43 ^C^ ± 8.48
8	0.05 ^H^ ± 0.00	3.00 ^HI^ ± 0.08	0.71 ^F^ ± 0.02	1.96 ^G^ ± 0.13	0.30 ^H^ ± 0.02	18.67 ^G^ ± 0.65	3.04 ^G^ ± 0.07	0.01 ^E^ ± 0.00	3.90 ^G^ ± 0.13	31.64 ^FG^ ± 1.21
9	0.14 ^E^ ± 0.00	24.17 ^B^ ± 1.06	26.90 ^C^ ± 0.97	1.99 ^G^ ± 0.07	0.70 ^E^ ± 0.02	0.98 ^I^ ± 0.03	223.73 ^B^ ± 7.52	0.01 ^E^ ± 0.00	0.34 ^B^ ± 0.02	278.96 ^C^ ± 7.79
10	0.16 ^D^ ± 0.01	18.55 ^D^ ± 0.74	1.11 ^F^ ± 0.04	1.09 ^G^ ± 0.04	0.55 ^FG^ ± 0.03	90.95 ^A^ ± 3.85	178.58 ^D^ ± 5.42	0.01 ^E^ ± 0.00	0.08 ^G^ ± 0.00	291.08 ^C^ ± 7.80

A–I—the mean with the same letters in the superscript did not differ significantly (*p* ≤ 0.01).

**Table 3:** The concentration of salicylic compounds in young shoots of the selected willow cultivars, the third 10-day period of May 2017 (µg/g DM). The numbers of cultivars as in Table 1.

Salicylic Compound						
Cultivars No.	Helicine	Salidroside	Saligenin	Salicin	Picein	Together
1	1.64 ^F^ ± 0.05	1.01 ^G^ ± 0.03	9.70 ^E^ ± 0.12	31.89 ^D^ ± 0.35	3.59 ^A^ ± 0.0.39	47.83 ^D^ ± 2.43
X_1_	2.24 ^C^ ± 0.03	0.20 ^E^ ± 0.01	56.08 ^A^ ± 1.41	102.33 ^A^ ± 2.62	2.35 ^C^ ± 0.07	163.20 ^A^ ± 3.65
2	1.95 ^E^ ± 0.02	0.62 ^H^ ± 0.00	16.55 ^D^ ± 0.21	90.72 ^B^ ± 2.27	1.14 ^D^ ± 0.02	110.98 ^C^ ± 3.11
4	1.02 ^H^ ± 0.03	0.58 ^H^ ± 0.02	2.93 ^G^ ± 0.03	17.18 ^F^ ± 0.21	0.74 ^EF^ ± 0.02	22.45 ^F^ ± 0.61
4	2.07 ^D^ ± 0.02	1.17 ^G^ ± 0.01	45.21 ^C^ ± 1.14	60.22 ^C^ ± 1.97	2.63 ^BC^ ± 0.03	111.30 ^C^ ± 3.68
5	2.96 ^A^ ± 0.04	1.93 ^E^ ± 0.02	6.89 ^F^ ± 0.03	24.25 ^E^ ± 0.30	1.11 ^DE^ ± 0.02	37.14 ^E^ ± 1.46
6	2.54 ^B^ ± 0.04	0.61 ^H^ ± 0.02	16.05 ^D^ ± 0.05	35.12 ^D^ ± 0.37	0.43 ^FG^ ± 0.02	54.75 ^D^ ± 2.37
X_2_	0.02 ^K^ ± 0.00	3.73 ^C^ ± 0.14	1.73 ^GH^ ± 0.05	1.97 ^H^ ± 0.06	0.20 ^G^ ± 0.01	7.65 ^H^ ± 0.22
X_3_	1.40 ^G^ ± 0.02	1.38 ^F^ ± 0.03	1.74 ^GH^ ± 0.04	11.23 ^G^ ± 0.32	0.46 ^FG^ ± 0.01	16.21 ^FG^ ± 0.48
7	0.81 ^I^ ± 0.01	3.91 ^B^ ± 0.04	0.85 ^I^ ± 0.01	4.08 ^G^ ± 0.05	0.43 ^FG^ ± 0.01	10.08 ^GH^ ± 0.32
8	1.00 ^H^ ± 0.02	0.39 ^I^ ± 0.01	53.82 ^B^ ± 1.02	63.18 ^C^ ± 0.99	2.87 ^B^ ± 0.03	121.26 ^B^ ± 4.03
9	0.54 ^J^ ± 0.01	2.78 ^D^ ± 0.03	5.63 ^F^ ± 0.14	4.96 ^H^ ± 0.07	0.52 ^FG^ ± 0.01	14.43 ^FGH^ ± 0.67
10	0.56 ^J^ ± 0.01	12.37 ^A^ ± 0.05	1.65 ^GH^ ± 0.02	1.24 ^H^ ± 0.03	0.77 ^EF^ ± 0.02	16.59 ^FG^ ± 0.56

A–I—the mean with the same letters in the superscript did not differ significantly (*p* ≤ 0.01).

**Table 4:** The total contents of phenolic acids, flavonoids and salicylate compounds in young stems of selected willow species, varieties and cultivars, spring of 2017 (µg/g DM). The numbers of cultivars as in Table 1.

Cultivars No.	Together
1.	182.42 ± 7.04
X_1_.	286.69 ± 12.78
2.	892.98 ± 35.27
3.	204.40 ± 8.31
4.	224.56 ± 9.23
5.	219.79 ± 8.15
6.	99.02 ± 4.90
X_2_.	98.30 ± 3.26
X_3_.	504.45 ± 22.61
7.	350.93 ± 16.56
8.	231.93 ± 10.37
9.	367.62 ± 14.24
10.	415.69 ± 17.49

**Table 5:** The number of individuals of deer animals found because of inventory in the spring of 2017 and 2019 in hunting districts where the browse plots were established.

Location of the Plot	Hunting Circuit	Year of Animal Inventory	Number of Deer (pcs)	Number of Fallow Deer (pcs)	Number of Roe Deer (pcs)	Total Cervids (pcs)
Bieczyny	211 211	2017 2019	200 175	5 12	330 265	535 452
Grzybno	210 210	2017 2019	107 98	30 19	330 264	467 381
Słonin	330	2017	25	80	550	655
	330	2019	23	80	500	603

**Table 6:** The Spearman’s rank correlation coefficients between the extent of foraging in the evaluated plots vs. specific salicylic compound content in willow. * Significant at *p* < 0.05.

Plot	Helicine	Salidroside	Saligenin	Salicin	Picein	Phenol’s Total
Total from plots (*n* = 90)	−0.21 *	0.21	−0.11	−0.22 *	−0.09	−0.10
Bieczyny (*n* = 30)	−0.27	0.17	0.04	−0.09	−0.12	0.08
Grzybno (*n* = 30)	−0.30	0.28	−0.01	−0.23	0.03	−0.08
Słonin (*n* = 30)	−0.38 *	0.15	−0.40 *	−0.41 *	−0.32	−0.26

**Table 7:** The Spearman’s rank correlation coefficients between the extent of foraging in the evaluated plots vs. concentration of selected phenolic acids in willow. * Significant at *p* < 0.05.

Plot	Protocatechic	p-Coumaric	Ferulic	Chlorogenic	Trans-Cinnamic	Synapinic	Total Phenolic Acids
Total from plots (*n* = 90)	−0.14	0.08	0.28 *	0.14	0.17	0.28 *	0.13
Bieczyny (*n* = 30)	−0.25	0.07	0.20	0.18	0.28	0.29	0.06
Grzybno (*n* = 30)	−0.13	0.25	0.39 *	0.02	0.35 *	0.38 *	0.35
Słonin (*n*= 30)	−0.37 *	−0.27	−0.24	0.42	−0.01	0.15	−0.27

## Data Availability

Not applicable.

## Supplementary Materials

The following are available online at https://www.mdpi.com/article/10.3390/biology10070612/s1, Table S1: Qualitative and quantitative analyses were carried out using the Multiple Reaction Monitoring (MRM) method for appropriate external standards; Table S2. The calibration curve (the range of 5–100 nM) was linear with a correlation coefficient of 0.97.
