# Supplementary material for: The Phenolic Compounds in the Young Shoots of Selected Willow Cultivars as a Determinant of the Plants’ Attractiveness to Cervids (Cervidae, Mammalia)
Source: Biology (Basel). 2021 Jul 2;10(7):612. doi: 10.3390/biology10070612 (PMC8301130; doi:10.3390/biology10070612)
Supplement: Supplementary file 1 [file biology-10-00612-s001.zip › S Tables.pdf]

Table S1. Qualitative and quantitative analyses were carried out using the Multiple Reaction Monitoring (MRM) method for appropriate external standards.

**Ion source conditions**	**Ionization mode**
	Negative
Nitrogen curtain gas	25 L/min
Collision gas	9 L/min
Ion spray source voltage	-4500 V
Temperature	350 °C
Nebulizer gas	35 L/min
Turbo gas	30 L/min

Table S2. The calibration curve (the range of 5-100 nM) was linear with a correlation coefficient of 0.97.

**Compound**	**Q1 (Da)**	**Q3 (Da)**	**Declastering potential (V)**	**Collision energy (V)**	**Collision cell exit potential (V)**
helicin	283.0	93.0	-70	-50	-12
salicin	285.0	123.0	-100	-45	-10
salidroside	299.1	89.0	-130	-19	-10
saligenin	123.0	105.1	-65	-19	-10
picein	297.0	135.2	-60	-26	-16
ferulic acid	193.0	134.1	-140	-24	-12
p-coumaric acid	163.0	119.0	-90	-20	-12
sinapic acid	223.0	208.1	-100	-21	-12
caffeic acid	179.0	135.0	-90	-25	-14
chlorogenic acid	353.2	191.1	-60	-33	-15
protocatechuic acid	153.0	109.0	-100	-10	-16
t-cinnamic acid	147.0	103.1	-145	-18	-12
vanillic acid	167.1	108.0	-100	-16	-14
isovanillic acid	167.0	152.0	-95	-15	-15
quercetin	301.1	151.1	-115	-35	-12
isorhamnetin	315.1	300.1	-125	-32	-25
kaempferol	285.0	93.0	-100	-50	-15
apigenin	269.0	117.0	-100	-50	-10
luteoin	285.1	133.1	-110	-40	-14
catechin	289.2	245.3	-100	-22	-16
naringenin	271.0	151.0	-100	-26	-11
prunin	433.2	271.1	-170	-27	-18
taxifolin	303.0	285.1	-95	-19	-21
